# Characterization of clonal immunoglobulin heavy V-D-J gene rearrangements in Chinese patients with chronic lymphocytic leukemia: Clinical features and molecular profiles

**DOI:** 10.3389/fonc.2023.1120867

**Published:** 2023-02-16

**Authors:** Xinyue Deng, Meilan Zhang, Jiachen Wang, Xiaoxi Zhou, Min Xiao

**Affiliations:** ^1^ Department of Hematology, Tongji Hospital, Tongji Medical College, Huazhong University of Science and Technology, Wuhan, Hubei, China; ^2^ Immunotherapy Research Center for Hematologic Diseases of Hubei Province, Wuhan, Hubei, China

**Keywords:** chronic lymphocytic leukemia, B-cell receptor, gene mutation, prognostic factors, next generation (deep) sequencing (NGS)

## Abstract

**Introduction:**

Several prognostic factors of chronic lymphocytic leukemia (CLL) have been identified, such as cytogenetic aberrations and recurrent gene mutations. B-cell receptor (BCR) signaling plays an important role in the tumorigenesis of CLL, and its clinical significance in predicting prognosis is also under study.

**Methods:**

Therefore, we assessed the already-known prognostic markers, immunoglobulin heavy chain (IGH) gene usage and the associations among these factors in 71 patients diagnosed with CLL in our center from October 2017 to March 2022. Sequencing of IGH gene rearrangements was performed using Sanger sequencing or IGH-based next-generation sequencing, and the results were further analyzed for distinct IGH/IGHD/IGHJ genes and the mutational status of the clonotypic IGHV (IGH variable) gene.

**Results:**

In summary, by analyzing the distribution of potential prognostic factors in CLL patients, we displayed a landscape of molecular profiles, confirmed the predictive value of recurrent genetic mutations and chromosome aberrations, and found that IGHJ3 was associated with favorable markers (mutated IGHV, trisomy 12), while IGHJ6 tended to correlate with unfavorable factors (unmutated IGHV, del17p).

**Discussion:**

These results provided an indication for IGH gene sequencing in predicting the prognosis of CLL.

## Introduction

Chronic lymphocytic leukemia (CLL) is the most prevalent adult leukemia in the Western world. The clinical course of CLL is highly heterogeneous, with the majority of cases identified as a relatively indolent type and a minority of cases marked by aggressive progress or malignant transformation despite intensive treatment (1). In recent decades, several predictive markers of CLL prognosis were revealed in succession. Clinical characteristics that may adversely influence the outcome of CLL include age (>65 years old), level of β-microglobulin (>3.5 mg/L) in serum, and Rai stage (I-IV) or Binet stage (B-C). Immunogenetic factors that may be involved in the prediction of prognosis included cytogenetic aberrations (del17p, del11q, del13q, and tri12), gene mutations (mutated *TP53*, *ATM*, *NOTCH1*, etc.), and unmutated status of the IGHV region (2).

Many studies have focused on IGHV, IGHD, and IGHJ gene usage or stereotyped major subsets of BCRs in CLL patients (3–5). BCR stereotypy refers to highly similar alignment of the complementarity determining region 3 (CDR3) region in the heavy (HCDR3) or light chain (LCDR3), which was found in approximately 41% of CLL patients. A total of 29 major subsets have been identified to date, each accompanied by several satellite subsets. CLL patients with BCR stereotypy showed distinct clinical profiles; for example, the subset #2 subgroup exhibited aggressive behavior independent of the mutational status of the clonotypic IGHV gene, while the IGHV4-34-containing subsets #4 and #16 had a more indolent progression. The prevalence of stereotyped BCR in CLL patients indicated a critical role of BCR signaling in the pathogenesis of this disease. The features of V(D)J rearrangements in Western CLL patients have been well studied and described; however, those in Chinese CLL patients are not clearly and systematically described. The main aim of the research was to describe IGHV, IGHD, and IGHJ genes and their correlation with other confirmed prognostic markers in Chinese CLL patients. This integrative demonstration could help better stratify patients and provide information about the mechanism of BCR signaling in CLL pathogenesis. Strong correlations between IGHV, IGHD, and IGHJ genes in CLL malignant clones and other different risk-stratified or prognostic biomarkers were well established in several large series of CLL patients (6–8). To verify and clarify these specific correlations in our cohort of Chinese CLL patients, we performed fluorescence *in situ* hybridization (FISH), karyotype analysis, and targeted next-generation sequencing (NGS) for gene mutations, combined with Sanger sequencing or NGS, to identify clonal B-cell immunoglobulin heavy chain (IGH) gene rearrangements and to assess the extent of somatic hypermutation (SHM) in the IGHV sequence in 71 CLL patients in our center. Then, we analyzed the possible associations and tried to summarize a landscape for subsequent mechanistic studies.

## Materials and methods

### Patients

This study included 71 CLL patients diagnosed by iwCLL criteria with results of V(D)J rearrangements in Tongji Hospital from October 2017 to March 2022 (9). A total of 71 peripheral blood (PB), bone marrow (BM) or lymph node (LN) samples were collected and underwent cytogenetic analysis, including 30 cases examined by karyotyping and 50 cases examined by FISH. Clinicopathologic features, including age (71/71), sex (71/71), and Rai stage or Binet stage (37/71), were retrospectively collected. Samples with incomplete or inconclusive results of V(D)J rearrangements were excluded from this study. All samples were collected after written informed consent was obtained in accordance with the Declaration of Helsinki.

### Morphology and immunophenotyping analysis

PB smears were stained using a standard Wright-Giemsa protocol and then prepared for manual 100-cell differential white blood cell count. BM aspirate smears were prepared for 200-cell differential white blood cell count, focusing on lymphocytes. The panel of monoclonal antibodies used in immunophenotyping analysis included CD5, CD10, CD11c, CD19, CD20, CD22, CD23, CD25, CD38, CD45, CD79b, FMC-7, CD2, CD3, CD4, CD7, CD8, CD56, CD10, and immunoglobulin κ and λ light chains (BD Biosciences).CLL scores were calculated based on the system proposed by Matutes et al. (10). Patients with a score of 4-5 were considered typical, while patients with a score of 3 were considered atypical. Patients with a score below 3 were excluded from this study.

### Conventional cytogenetics

Tests for cytogenetics were performed in a total of 30 cases. Cells in collected samples were cultured at 1×10^6^ cells/mL in the presence of CpG-oligonucleotide DSP30 (2 μmol/L) and IL-2 (0.2 μg/mL) for 72 hours. The banding process was then performed following standard protocols. When possible, at least 20 metaphases were analyzed, and a number of metaphases were marked in the report. The karyotype was analyzed based on the International System for Human Cytogenetic Nomenclature (ISCN 2016). An abnormal clone was identified by structural aberrations, including chromosome gains observed in at least two different metaphases or chromosome losses observed in at least three different metaphases. A complex karyotype was defined as a clone that possesses at least three independent abnormalities at the same time.

### FISH

Interphase FISH was performed without sorting on 200 cells from 50 cases from PB, BM or LN samples using a commercial probe panel (MetaSystems, Altlussheim, Germany). Panels included *TP53/CEP17* (del17p), *ATM* (del11q), trisomy 12, *D13S29* (del13q), and translocations between *IGH* and the partner gene (*IGH/CCND1*). The t(11;14)(q13;q12) *IGH/CCND1* dual- color dual- fusion translocation probe was used to identify and rule out cases of mantle cell lymphoma (MCL). Cases with del13q alone or absence of FISH aberrations were categorized as a favorable group according to the Dohner FISH classification (11), while cases with del11q or del17p were considered unfavorable. The group of patients with trisomy 12 was considered to have an intermediate prognosis. The probe cutoff values were set as 5% for the deletion probe, 3% for the trisomy probe, and 1% for the dual-color dual- fusion probe.

### Sanger sequencing and IGH-based NGS of the V(D)J rearrangements

Sequencing of IGH gene rearrangements was performed in all 71 cases. Sanger sequencing of IGH gene rearrangements was performed in 58 cases, and NGS of IGH gene rearrangements was performed in the other 13 cases. Sanger sequencing and IGH-based NGS were performed on fresh, frozen or FFPE samples acquired from PB, BM or LNs (**Supplementary Table 1**). In the Sanger sequencing process, PCR amplification of IGHV-IGHD-IGHJ rearrangements was performed using either genomic DNA (gDNA) or cDNA as described in the InVivoScribe IGH Somatic Hypermutation Assay v2.0 - ABI Fluorescence Detection. Sequence information was analyzed based on direct sequencing of both strands using the IMGT databases and the IMGT/V-QUEST tool (http://www.imgt.org). Only productive rearrangements were further evaluated for parse, reorganization, and output. Information was sorted and reported as Ig gene repertoires, VH CDR3 length, exact amino acid sequence and SHM. In the IGH-based NGS process, the CLL clonotype was established in the tumor sample using locus-specific primer sets for IGHV, IGHD, and IGHJ rearrangements and the MiSeq Illumina platform (LymphoTrack Dx IGHV Leader Somatic Hypermutation Assay-Miseq). The output form of the results was then further analyzed based on the international ImMunoGeneTics (IMGT) information system to identify the exact IGHV-D-J sequence and the corresponding frequency, as previously described (12). In a patient sample, a clonotype present at a frequency of higher than 5% of all rearranged V(D)J sequences was identified as a malignant clone. The clone present at the highest frequency in the tumor sample was named the “calibrating” or “index” clone. The mutational status of the clonotypic IGHV gene was defined as follows: 1) a clone carrying IGHV sequences that exhibited ≥98% homology compared to germline sequences was considered unmutated; 2) a clone carrying IGHV sequences that exhibited <98% homology compared to the germline sequences was considered mutated; and 3) if there were double rearrangements, the mutational status of the IGHV gene in a CLL patient was determined by the suggested double rearrangement SHM interpretation criteria (ERIC) (13). Major-subset stereotyped BCRs were identified by the Arrest/assign Tool (), in which the basic algorithms were established according to the study by Agathangelidis et al. (14).

### Targeted NGS

Genetic mutations were detected by targeted NGS panels in 46 cases. Total DNA from PB, BM or LN samples was extracted for targeted NGS to detect gene mutations. Targeted NGS was also performed using the Ion Torrent/Illumina NextSeq 550Dx platform. The panels used are summarized in **Supplementary Table 2**. To minimize potential artifacts, only somatic variants with a PASS filter, read depth DP≥500 and VAF≥2% were retained. The filter criteria included 1) nonsynonymous mutations in the exonic region, splicing mutations, and UTR mutations in NOTCH1; 2) allele frequency of the mutated gene < 0.01 in the global population in the 1000G, ExAC, and gnomAD databases; and 3) other gene mutations that were proven to have clinical significance. Particular attention was given to mutated *TP53, NOTCH1, MYD88, SF3B1, ATM*, and *MYC.*

### Statistical analysis

Statistical analysis was performed in different groups of patients classified by distinct tests. Chi square and Fisher’s exact tests were used to assess the associations between categorical variables. One-way ANOVA was used to assess intergroup differences. Statistically, a significant difference was confirmed when *P*<0.05 in a two-sided test. All analyses were performed using SPSS 23.0.

## Results

### Baseline characteristics of patients

Seventy-one patients met the inclusion criteria in our study. IGH gene sequencing was performed in all of these patients for the collection and analysis of patterns in V(D)J rearrangements and the extent of SHM. The median age was 61 years, with a range from 36 years to 82 years. Twenty-one females and 50 males were included. The ratio of gender was approximately 1:2, similar to previous reports (15). Among the 71 patients, 37 were newly diagnosed with CLL, in whom the Rai stage and Binet stage were assessed. Of the 37 patients, 9 were diagnosed with CLL Binet A stage, and 28 were diagnosed with Binet B-C stage. Only 2 patients were assessed as Rai stage 0, while the other 35 were assessed as Rai stage I-IV. Molecular features included the mutational status of the clonotypic IGHV gene, FISH aberrations, karyotyping, and immunophenotype analysis. Twenty-one (29.58%) of 71 patients carried an unmutated IGHV region, while 48 (67.61%) of 71 patients possessed a mutated IGHV region. This finding again verified that the frequency of mutated IGHV in Chinese CLL patients (M:U≈2:1) was higher than that in Western CLL patients (M:U≈1:1) (16). Among 50 patients in whom FISH was performed, 6 carried a del17p aberration, 3 carried an 11q deletion, 6 possessed an extra chromosome 12, 22 carried a del13q aberration, and 15 did not have any FISH aberrations. Karyotyping was performed in 33 patients, including 28 with < 3 aberrations and 5 with a complex karyotype. All baseline characteristics of the patients are summarized in **Table 1**.

**Table 1:** Summary of the clinical and molecular characteristics at baseline.

Variable	Patients
**Patients, n**	71
**Median age (range), y**	61 (36-82)
**Sex, n (%)**	
Female	21 (29.58)
Male	50 (70.42)
**Binet Stage, n (%)**	
A	9 (12.68)
B	17 (23.94)
C	11 (15.49)
Unknown	34 (47.89)
**Rai Stage, n (%)**	
**0**	2 (2.82)
I	13 (18.31)
II	9 (12.68)
III	4 (5.63)
IV	9 (12.68)
Unknown	34 (47.89)
**IGHV status, n (%)**	
Unmutated	21 (29.58)
Mutated	48 (67.61)
Inconclusive	2 (2.81)
**Hierarchical model by FISH, n (%)**	
Del(17p)	6 (8.45)
Del(11q)	3 (4.23)
Tri(12)	6 (8.45)
Del(13q)	22 (30.99)
None	15 (21.13)
Unknown	21 (29.58)
**Conventional karyotype, n (%)**	
<3 aberrations	28 (39.44)
≥3 aberrations (complex karyotype)	5 (7.04)
Unknown	38 (53.52)

### IGHV, IGHD, and IGHJ gene usage in CLL patients

**Figure 1:**
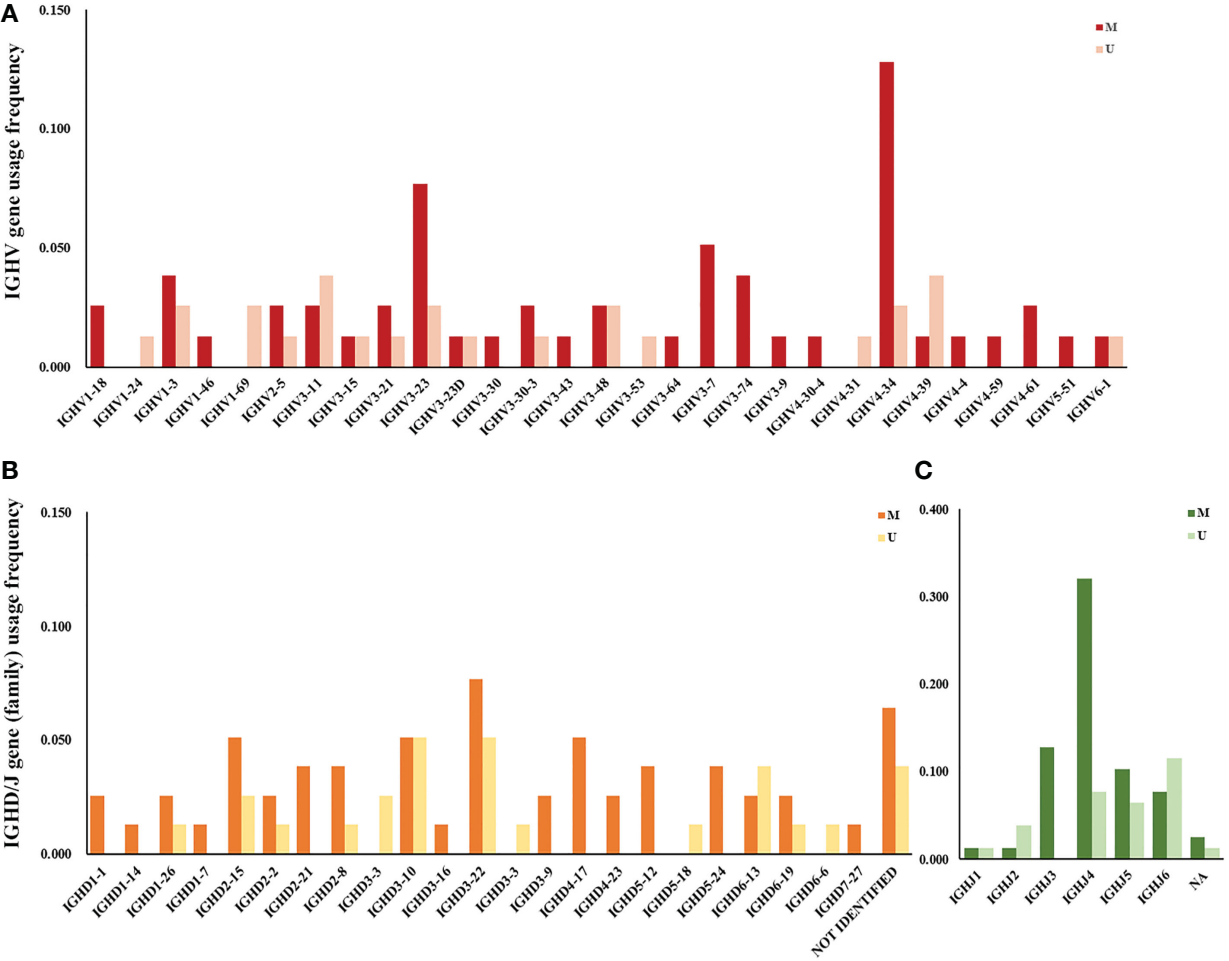
IGHV, IGHD, and IGHJ gene (subgroup) usage frequency in Chinese CLL patients. **(A)** V subgroup/gene usage frequency in Chinese CLL patients. Red columns represent cases with mutated IGHV regions, and pink columns represent cases with unmutated IGHV regions. **(B)** D subgroup/gene usage frequency in Chinese CLL patients. Orange columns represent cases with mutated IGHV regions, and yellow columns represent cases with unmutated IGHV regions. **(C)** J subgroup/gene usage frequency in Chinese CLL patients. Dark green columns represent cases with mutated IGHV regions, and light green columns represent cases with unmutated IGHV regions.

The frequency of IGHV, IGHD, and IGHJ gene usage is summarized in **Table 2**. Similar to previous studies (17), among IGHV genes used in CLL cases, the IGHV3 subgroup was most frequently used, followed by IGHV4 and IGHV1. IGHV4-34, IGHV3-23, IGHV3-11, IGHV1-3, and IGHV4-39 were the most frequently selected genes (**Figure 1A**). For IGHD genes, the IGHD3 and IGHD2 subgroups were preferentially selected. The IGHD3-22, IGHD3-10, and IGHD2-15 genes were the most frequently used genes (**Figure 1B**). Among the IGHJ genes, the IGHJ4 gene was most frequently used, followed by IGHJ3 and IGHJ5 (**Figure 1C**). Next, we assessed the associations between IGHV, IGHD, and IGHJ genes used in CLL patients and the mutational status of the clonotypic IGHV gene by Fisher’s exact test. As shown in **Table 2**, patients using the IGHJ3 gene were all mutated with statistical significance (10/10, *P*=0.0257<0.05), while the cases using the IGHJ6 gene preferentially possessed an unmutated IGHV region (9/15, *P*=0.0146<0.05). The significant overrepresentation of IGHJ6 in cases with an unmutated IGHV region was previously reported (18). IGHJ4 also tended to co-occur with the mutated IGHV region, but the correlation was not significant (25/31, *P*=0.0817). CLL patients carrying a member of the IGHD3 gene subgroup tended to possess an unmutated IGHV region, but it was not significant. Additionally, clones carrying genes from IGHJ2 seemed to preferentially pair with an unmutated IGHV region (3/4).

**Table 2:** IGHV, IGHD subgroups, and IGHJ genes used in CLL patients in our study.

IGH Subgroups and Genes	Number and frequency of IGHV mutated cases, n (%)	Number and frequency of IGHV unmutated cases, n (%)	*P* value	Number and frequency of total patients, n (%)
**Total**	53 (100.00)	25 (100.00)		78 (100.00)
**IGHV1**	6 (11.32)	5 (20.00)	0.3163	11 (14.10)
**IGHV2**	2 (3.77)	1 (4.00)	>0.9999	3 (3.85)
**IGHV3**	27 (50.94)	12 (48.00)	>0.9999	39 (50.00)
IGHV3-11	2 (3.77)	3 (12.00)	0.3203	5 (6.41)
IGHV3-21	2 (3.77)	1 (4.00)	>0.9999	3 (3.85)
IGHV3-23	6 (11.32)	2 (8.00)	>0.9999	8 (10.26)
IGHV3-48	2 (3.77)	2 (8.00)	0.5894	4 (5.13)
Other	15 (28.30)	4 (16.00)		19 (24.36)
**IGHV4**	16 (30.19)	6 (24.00)	0.7880	22 (28.21)
IGHV4-34	10 (18.87)	2 (8.00)	0.3186	12 (15.38)
IGHV4-39	1 (1.89)	3 (12.00)	0.0943	4 (5.13)
Other	5 (9.43)	1 (4.00)		6 (7.69)
**IGHV5**	1 (1.89)	0 (0.00)	>0.9999	1 (1.28)
**IGHV6**	1 (1.89)	1 (4.00)	0.5411	2 (2.56)
**IGHD1**	6 (11.32)	1 (4.00)	0.4192	7 (8.97)
**IGHD2**	12 (22.64)	4 (16.00)	0.5637	16 (20.51)
IGHD2-15	4 (7.55)	2 (8.00)	>0.9999	6 (7.69)
Other	8 (15.09)	2 (8.00)		10 (12.82)
**IGHD3**	13 (24.53)	11 (44.00)	0.1149	24 (30.77)
IGHD3-10	4 (7.55)	4 (16.00)	0.2604	8 (10.26)
IGHD3-22	6 (11.32)	4 (16.00)	0.7183	10 (12.82)
Other	3 (5.66)	3 (12.00)		6 (7.69)
**IGHD4**	6 (11.32)	0 (0.00)	0.1689	6 (7.69)
**IGHD5**	6 (11.32)	1 (4.00)	0.4192	7 (8.97)
**IGHD6**	4 (7.55)	5 (20.00)	0.1363	9 (11.54)
IGHD6-13	2 (3.77)	3 (12.00)	0.3203	5 (6.41)
Other	2 (3.77)	2 (8.00)		4 (5.13)
**IGHD7**	1 (1.89)	0 (0.00)	>0.9999	1 (1.28)
**N/A**	5 (9.43)	3 (12.00)		8 (10.26)
**IGHJ1**	1 (1.89)	1 (4.00)	0.5411	2 (2.56)
**IGHJ2**	1 (1.89)	3 (12.00)	0.0943	4 (5.13)
**IGHJ3**	10 (18.87)	0 (0.00)	0.0257*	10 (12.82)
**IGHJ4**	25 (47.17)	6 (24.00)	0.0817	31 (39.74)
**IGHJ5**	8 (15.09)	5 (20.00)	0.7458	13 (16.67)
**IGHJ6**	6 (11.32)	9 (36.00)	0.0146*	15 (19.23)
**N/A**	2 (3.77)	1 (4.00)		3 (3.85)

The data was analyzed using Fisher’s exact test with α<0.05 (two-sided). The asterisk (*) indicates that there is statistical significance (P value<0.05).

The associations among IGHV, D, and J genes (subgroups) used in CLL patients and the mutational status of the clonotypic IGHV gene were analyzed using Fisher’s exact test with a<0.05 (two-sided). The asterisk (*) indicates that there is statistical significance (P value <0.05).

Bold values indicate statistical significances (P value < 0.05).N/A, Not applicable.

Next, we investigated the correlations between the IGHV, IGHD, and IGHJ genes used in our cohort and other molecular aberrations using the chi-square test or Fisher’s exact test (**Table 3**). The molecular aberrations involved included the presence of trisomy 12, del(11q), del(13q), del(17p), complex karyotype, mutated TP53 gene, and unmutated IGHV region. No significant difference was found in the analysis involving IGHV gene subgroups. Cases with BCRs rearranged using genes in the IGHV1 subgroup tended to carry a deletion of 11q, but the difference among IGHV groups was not significant (*P*=0.0592). Cases with BCRs rearranged using genes in the IGHD3 and IGHD5 subgroups carried significantly more del13q aberrations than the IGHD2 group (intergroup: *P*=0.0292<0.05; IGHD3 and IGHD2: *P*=0.039<0.05; IGHD5 and IGHD2: *P*=0.035 <0.05). Cases with BCRs using genes from the IGHD3 or IGHD6 subgroups tended to pair with unmutated IGHV. IGHD6-expressing patients also exhibited a higher frequency of complex karyotypes than other IGHD groups. Surprisingly, 3 of the detected aberrations or statuses exhibited statistically significant differences among the IGHJ genes. Patients using genes in the IGHJ3 group exhibited significantly more trisomy 12 (3/6) and paired with unmutated IGHV with a significantly lower frequency compared with IGHJ4 (1/26, *P*=0.0149 < 0.05) and IGHJ5 (0/10, *P*=0.0357 <0.05) genes. IGHJ5 and trisomy 12 seemed to be mutually exclusive. IGHJ6-expressing patients possessed significantly (*P*=0.0329 <0.05) more del17p (3/8) aberrations than the IGHJ4 (1/26) group. However, the specific clinical significance of IGHJ genes themselves used in CLL patients was not clearly demonstrated. The presence of trisomy 12 and a mutated IGHV region were considered favorable prognostic markers in some studies (19, 20); thus, we propose that the use of IGHJ3 genes possibly correlates with better outcomes in CLL. Similarly, the use of IGHD3 subgroups was correlated with del13q alone, which may indicate a better prognosis. Conversely, the use of IGHJ6 genes tended to co-occur with del17p and was possibly associated with TP53 mutation (**Table 3**), which may indicate an unfavorable outcome.

**Table 3:** Correlation of IGH subgroups/genes and molecular prognostic markers.

IGH Subgroups and Genes	Molecular prognostic markers, n/number of cases with specific IGH gene subgroup/genes (%)						
	FISH (n=55)				Chr. (n=47)	Mutation (n=52)	IGHV (n=78)
	Tri (12)	Del (11q)	Del (13q)	Del (17p)	CK	TP53	U
**Total, n**	7	4	22	6	6	6	25
**IGHV1**	0/7 (0.00)	2/7 (28.57)	3/7 (42.86)	2/7 (28.57)	2/7 (28.57)	2/9 (22.22)	5/11 (45.45)
**IGHV2**	0/3 (0.00)	0/3 (0.00)	1/3 (33.33)	0/3 (0.00)	0/2 (0.00)	0/1 (0.00)	1/3 (33.33)
**IGHV3**	3/30 (10.00)	0/30 (0.00)	14/30 (46.67)	2/30 (6.67)	1/21 (4.76)	3/30 (10.00)	12/39 (30.77)
**IGHV4**	4/14 (28.57)	2/14 (14.29)	3/14 (21.43)	1/14 (7.14)	2/15 (13.33)	0/10 (0.00)	6/22 (27.27)
**IGHV5**	0/0 (0.00)	0/0 (0.00)	0/0 (0.00)	0/0 (0.00)	0/0 (0.00)	0/1 (0.00)	0/1 (0.00)
**IGHV6**	0/1 (0.00)	0/1(0.00)	0/1 (0.00)	1/1 (100.00)	1/2 (50.00)	1/1 (100.00)	1/2 (50.00)
***P* value**	0.3756	0.0592	0.5083	0.0744	0.1832	0.1322	0.8492
**IGHD1**	0/4 (0.00)	0/4 (0.00)	1/4 (25.00)	0/4 (0.00)	0/4 (0.00)	1/5 (20.00)	1/7 (14.29)
**IGHD2**	1/9 (11.11)	2/9 (22.22)	1/9 (11.11)	1/9 (11.11)	2/9 (22.22)	1/7 (14.29)	4/16 (25.00)
**IGHD3**	1/19 (5.26)	1/19 (5.26)	11/19 (57.89)	2/19 (10.53)	1/16 (6.25)	1/19 (5.26)	11/24 (45.83)
**IGHD4**	2/4 (50.00)	0/4 (0.00)	3/4 (75.00)	0/4 (0.00)	0/5 (0.00)	0/6 (0.00)	0/6 (0.00)
**IGHD5**	0/7 (0.00)	1/7 (14.29)	5/7 (71.43)	0/7 (0.00)	0/5 (0.00)	0/3 (0.00)	1/7 (14.29)
**IGHD6**	2/7 (28.57)	0/7 (0.00)	1/7 (14.29)	3/7 (42.86)	3/6 (50.00)	3/8 (37.5)	5/9 (55.56)
**IGHD7**	0/1 (0.00)	0/1 (0.00)	0/1 (0.00)	0/1 (0.00)	0/0 (0.00)	0/0 (0.00)	0/1 (0.00)
**N/A**	1/4 (25.00)	0/4 (0.00)	1/4 (25.00)	0/4 (0.00)	0/3 (0.00)	0/4 (0.00)	3/8 (37.50)
***P* value**	0.1291	0.6836	0.0292*	0.3109	0.1057	0.1992	0.1251
**IGHJ1**	1/2 (50.00)	0/2 (50.00)	0/2 (0.00)	1/2 (50.00)	0/2 (0.00)	1/1 (100.00)	1/2 (50.00)
**IGHJ2**	0/1 (0.00)	1/1 (100.00)	1/1 (100.00)	0/1 (0.00)	0/1 (0.00)	0/3 (0.00)	3/4 (75.00)
**IGHJ3**	3/6 (50.00)	0/6 (0.00)	3/6 (50.00)	0/6 (0.00)	0/5 (0.00)	0/5 (0.00)	0/10 (0.00)
**IGHJ4**	1/26 (3.85)	2/26 (7.69)	12/26 (46.15)	1/26 (3.85)	3/20 (15.00)	2/25 (8.00)	6/31 (19.35)
**IGHJ5**	0/10 (0.00)	1/10 (10.00)	5/10 (50.00)	1/10 (10.00)	1/9 (11.11)	0/7 (0.00)	5/13 (38.46)
**IGHJ6**	1/8 (12.50)	0/8 (0.00)	1/8 (12.50)	3/8 (37.50)	2/6 (33.33)	3/9 (33.33)	9/15 (60.00)
**N/A**	1/2 (50.00)	0/2 (0.00)	0/2 (0.00)	0/2 (0.00)	0/1 (0.00)	0/2 (0.00)	1/3 (33.33)
***P* value**	0.0129*	0.1926	0.2904	0.0458*	0.7345	0.0596	0.0021**

The data was analyzed using Fisher’s exact test with α<0.05 (two-sided). The asterisk (*) indicates that there is statistical significance (P value<0.05). P value < 0.05 indicates significant inter-group difference. * P<0.05. **P<0.01. Chr. Chromosome; CK, complex karyotype; tri, trisomy; del, deletion.

Molecular prognostic markers included FISH aberrations (trisomy 12/Tri12, deletion of 11q/Del11q, deletion of 13q/Del13q, deletion of 17p/Del17p), the aberrant karyotype (complex karyotype/CK), TP53 mutation, and unmutated IGHV region. The intergroup differences were analyzed using Fisher’s exact test with a<0.05 (two-sided). The asterisk (*) indicates that there is statistical significance (P value<0.05). P value < 0.05 indicates significant inter-group difference. * P<0.05. **P<0.01. Chr., chromosome. U, unmutated.Bold values indicate statistical significances (P value < 0.05).N/A, Not applicable.

The analysis of associations between clinical features and IGH gene subgroup usage was also performed, but no significant difference was found among the IGHV, IGHD, and IGHJ subgroups (**Supplementary Table 3**). The intergroup analysis indicated significant differences in sex among IGHV groups and in Rai stage among IGHJ groups; however, when compared in pairs, there was no statistically significant difference.

**Figure 2:**
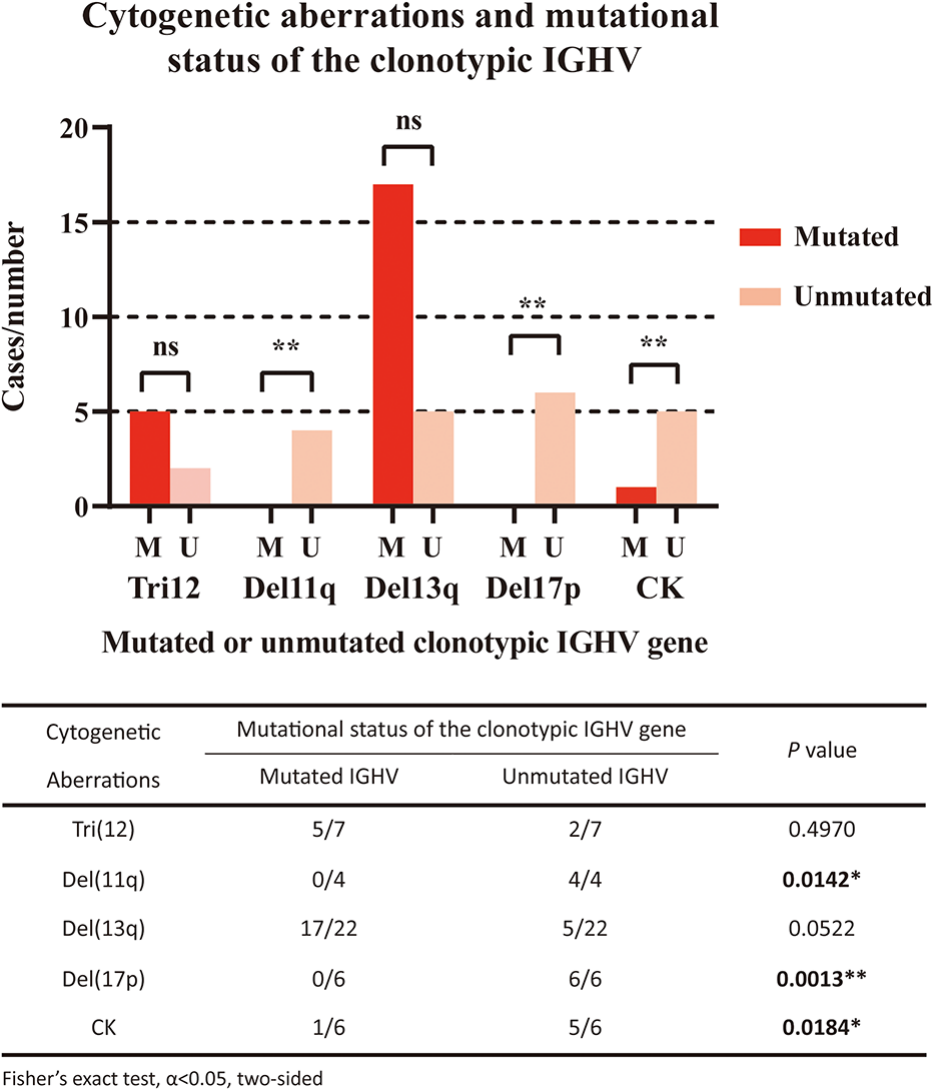
Correlation of cytogenetic aberrations and mutational status of the clonotypic IGHV gene. The five cytogenetic aberrations detected included FISH aberrations (trisomy 12/Tri12, deletion of 11q/Del11q, deletion of 13q/Del13q, deletion of 17p/Del17p) and the aberrant karyotype (complex karyotype/CK). In the right panel, red columns represent cases with mutated IGHV regions, while pink columns represent cases with unmutated IGHV regions. The statistical analysis was performed using Fisher’s exact test, α<0.05 (two-sided). Ns, nonsignificant. *P<0.05. ** *P*<0.01.

We also analyzed the correlations between the mutational status of the clonotypic IGHV gene and cytogenetic aberrations (**Figure 2**). Del11q (4/4, *P*=0.0142) and del17p (6/6, *P*=0.0013) were significantly associated with the unmutated status of the IGHV region, which was in accordance with the fact that these factors represented a worse prognosis. Del13q alone was correlated with mutated IGHV status, but not significantly (17/22, *P*=0.0522), and both markers were considered favorable. Trisomy 12 was equally distributed in the IGHV-mutated and IGHV-unmutated groups. A complex karyotype was also significantly associated with the unmutated IGHV region (5/6, *P*=0.0184).

**Figure 3:**
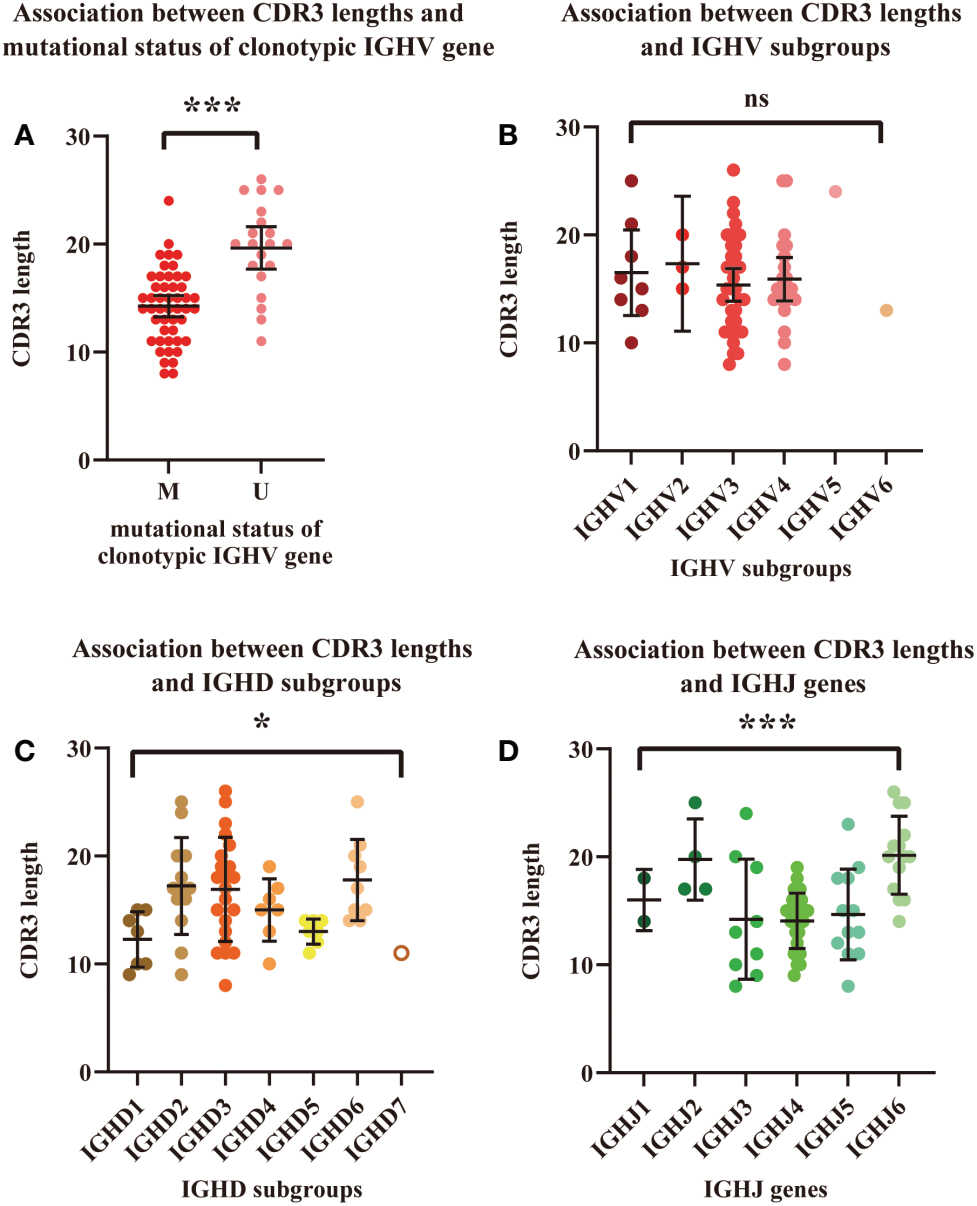
Association between HCDR3 length and mutational status of the clonotypic IGHV gene. **(A)** Association between HCDR3 length and IGHV mutational status. Red dots represent cases with mutated IGHV regions, and pink dots represent cases with unmutated IGHV regions. **(B)** Association between HCDR3 length and the IGHV subgroups used. **(C)** Association between HCDR3 length and the IGHD subgroups used. **(D)** Association between HCDR3 length and the IGHJ genes used. The statistical analysis was performed using Student’s t test, α<0.05 (two-sided). Ns, nonsignificant. * *P*<0.05. ***P<0.001.

The correlation between the length of HCDR3 and the mutational status of the clonotypic IGHV gene was analyzed (n=68) by Student’s t test (**Figure 3A**). Patients with unmutated IGHV had a significantly longer average length of the HCDR3 region than those with mutated IGHV (19.65vs. 14.25, *P*<0.0001). Differences in HCDR3 length among IGHV, IGHD, and IGHJ subgroups were also calculated (**Figures 3B–D**). Significant differences in HCDR3 lengths among IGH subgroups were found in the analysis of IGHD (*P*=0.0228 < 0.05) and IGHJ (*P*<0.0001) genes. Patients using genes in the IGHD2 (mean: 17.23), IGHD3 (mean: 16.91) and IGHD6 (mean: 17.78) subgroups had significantly longer average lengths of HCDR3 compared with those using genes in the IGHD1 (mean: 12.29) and IGHD5 (mean: 13.00) subgroups. Similarly, patients using genes in IGHJ2 (mean: 19.75) and IGHJ6 (mean: 20.14) had significantly longer average lengths of HCDR3 than those using genes in IGHJ1 (mean: 16.00), IGHJ3 (mean: 14.22), IGHJ4 (mean: 14.07), and IGHJ5 (mean: 14.67). However, no significant difference was found in the analysis of IGHV subgroups.

CLL clones were also marked by BCR stereotypy. However, filtered by the Arrest/assign Tool, there were only two patients that carried a major-subset stereotyped BCR in our cohort. The two stereotyped BCRs, realigned IGHV4-4/IGHD5-24/IGHJ4 and IGHV4-39/IGHD6-13/IGHJ5, matched major subset #77 and major subset #8, respectively. The ratio between mutated versus unmutated IGHV genes possibly influenced the frequency of stereotyped BCRs, as previously reported (6). However, this ratio in our cohort was similar to that in previous studies. Other factors, such as inheritance, ethnicity, and regional differences, may contribute to the absence or at least the low frequency of stereotyped BCRs in this study.

### Landscape of gene mutations in CLL

**Figure 4:**
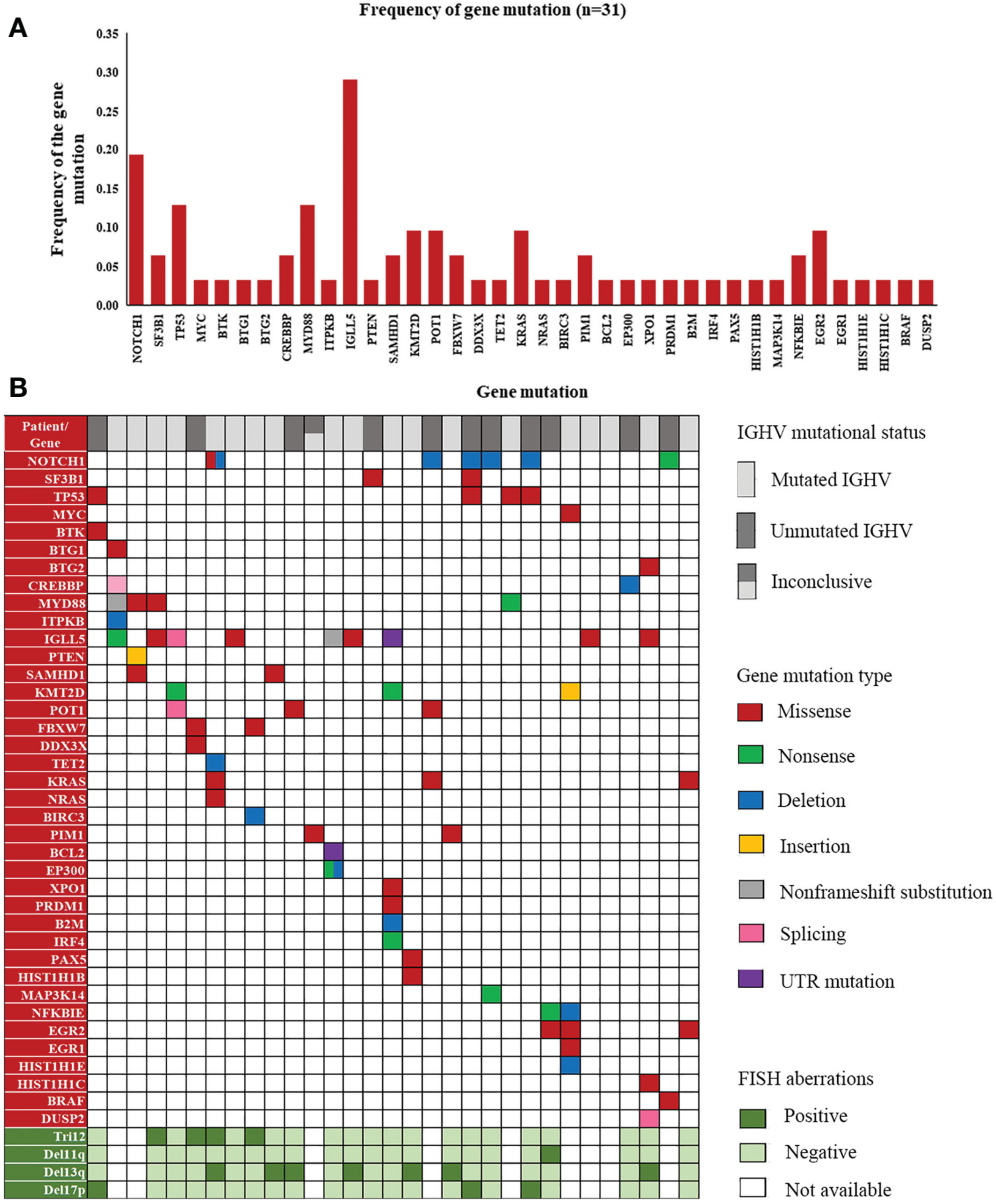
A landscape of gene mutations detected in our patients. **(A)** Frequency of gene mutations in cases detected by Panels 4 and 5. **(B)** Distribution of mutated genes with corresponding mutation types and FISH aberrations in the cases detected by Panels 4 and 5.

Screening for gene mutations was performed using targeted NGS. Five different gene panels were used due to the new discovery of genes involved in lymphoid malignancies from 2017 to 2022 (**Supplementary Table 2**). A total of 74 gene mutations were detected in 52 cases. Genes with the 10 highest frequencies of mutations were as follows: *IGLL5* (29.03%), *NOTCH1* (15.00%), *MYD88* (11.11%), *TP53* (8.70%), *POT1* (9.68%), *KMT2D* (9.68%), *EGR2* (9.68%), *KRAS* (8.57%), *NFKBIE* (6.25%), *SAMHD1* (8.00%), and *SF3B1* (5.00%) (**Supplementary Table 4**). Since the coverage of Panel 1 (genes: 3), Panel 2 (genes: 40) and Panel 3 (genes: 108) was incomplete, **Figure 4A** shows the frequency of gene mutations detected only by Panel 4 (genes: 129) and Panel5 (genes: 157). The distribution of mutated genes detected using Panel 4 or 5 and the corresponding mutation type are summarized in **Figure 4B**.

We next investigated the correlations between the number of mutated genes and other prognostic markers, but no significant correlation was identified in any groups classified based on different numbers of mutated genes (**Supplementary Tables 5**, **6**). The correlation between IGH gene subgroups used in CLL clones and the number of mutated genes was also not significant (**Supplementary Table 7**).

The genes with the 4 highest mutation frequencies were particularly considered, including *IGLL5, NOTCH1, MYD88*, and *TP53*. The association between these four mutated genes in CLL and molecular prognostic factors (n=22 in the analysis of FISH results, n=22 in the analysis of karyotyping results, and n=31 in the analysis of unmutated IGHV) was analyzed (**Table 4**). *NOTCH1* mutation and *TP53* mutation were significantly associated with del17p (*P*=0.0452 < 0.05; *P*=0.0008 < 0.05). *TP53* mutation was also found to correlate with complex karyotype (*P*=0.0452 < 0.05) and unmutated IGHV region (*P*=0.1141, insignificant). Mutated *NOTCH1* was significantly related to the unmutated IGHV region (*P*=0.0095 < 0.05). These results further confirmed the adverse effects of mutated *NOTCH1*, mutated *TP53*, an unmutated IGHV region, and a complex karyotype in CLL. Conversely, the *IGLL5* mutation with the highest frequency in our cohort was significantly associated with a mutated IGHV gene (9/9). Moreover, most patients with IGLL5 mutations did not have other known driving aberrations, such as *NOTCH1* mutations, *SF3B1* mutations, or *ATM* mutations. A previous study alsodemonstrated the unique features of cases carrying *IGLL5* mutations, including prominence in lower-risk mutated CLL, off-target activation-induced cytidine deaminase (AID) activity, and gene mutations previously undescribed (21). *IGLL5* was also demonstrated to be frequently mutated in B-cell malignancies, such as Hodgkin’s lymphoma (22), diffuse large B-cell lymphoma (DLBCL) (23), multiple myeloma (MM) (24), and CLL with translocations involving IGH (25). However, the exact mechanism of mutated *IGLL5* in the tumorigenesis of B-cell malignancies is not yet clear. Our findings may suggest that mutated *IGLL5* is correlated with lower-risk CLL pathogenesis.

**Table 4:** Correlation of specific gene mutations and molecular prognostic markers.

Mutated genes	Molecular prognostic markers, n (number of certain cases), *P*						
	FISH aberrations (positive or negative)					Karyotype (CK or other)	IGHV status (Unmutated or mutated)
	Tri (12)	Del (11q)	Del (13q)	Del (17p)	negative	CK	Unmutated
**IGLL5**	1, >0.9999	0, >0.9999	2, >0.9999	0, 0.5227	4, 0.3426	1, >0.9999	0, 0.0116*
**NOTCH1**	1, >0.9999	0, >0.9999	1, >0.9999	2, 0.0452*	1, >0.9999	1, 0.5107	5, 0.0095**
**MYD88**	1, 0.1600	0, >0.9999	0, >0.9999	0, >0.9999	0, >0.9999	0, >0.9999	0, 0.2752
**TP53**	0, >0.9999	0, >0.9999	0, 0.2379	3, 0.0008***	0, 0.5409	2, 0.0452*	3, 0.1141

The data was analyzed using the Fisher’s exact test with α<0.05 (two-sided). The asterisk (*) indicates that there is statistical significance (P value <0.05). * P<0.05. **P<0.01. ***P<0.001.

The associations among specific gene mutations and cytogenetic aberrations, karyotyping, and IGHV mutational status were analyzed using Fisher’s exact test with a<0.05 (twosided). The asterisk (*) indicates that there is statistical significance (P value <0.05). * P<0.05. **P<0.01. ***P<0.001. Tri12, trisomy 12. Del11q, deletion of 11q. Del13q, deletion of 13q. Del17p, deletion of 17p. CK, complex karyotype.Bold values indicate statistical significances (P value < 0.05).

The associations between the four mutated genes and baseline clinical features were also analyzed, but no significant correlations were found (**Supplementary Table 8**).

### Associations between specific gene mutations and IGH gene subgroups used in CLL

We then analyzed the association between the four gene mutations *(IGLL5, NOTCH1, MYD88*, and *TP53*) and the corresponding IGH gene subgroups used in these patients (**Table 5**). Patients using IGHJ4 genes tended to carry significantly fewer *NOTCH1* mutations than those using other IGHJ genes (intergroup: *P*=0.0231< 0.05). Patients using IGHV4 subgroups tended to carry more *IGLL5* and *MYD88* mutations, but the difference among IGHV subgroups was not significant.

**Table 5:** Correlation of specific mutated genes and usage of IGHV, IGHD subgroups and IGHJ genes.

IGHV Subgroups and Genes	Mutated genes, n (cases with mutated genes/cases with specific IGHV subgroup/gene, %)			
	IGLL5 (n=31)	NOTCH1 (n=40)	MYD88 (n=35)	TP53 (n=46)
**Total, n**	9	6	4	4
**IGHV1**	1/5 (20.00)	1/6 (16.67)	0/6 (0.00)	2/8 (25.00)
**IGHV2**	0/0 (0.00)	0/1 (0.00)	0/1 (0.00)	0/1 (0.00)
**IGHV3**	3/18 (16.67)	4/23 (17.39)	1/20 (5.00)	2/26 (7.69)
**IGHV4**	4/7 (57.14)	1/9 (11.11)	2/7 (28.57)	0/10 (0.00)
**IGHV5**	1/1 (100.00)	0/5 (0.00)	1/1 (100.00)	0/1 (0.00)
**IGHV6**	0/0 (0.00)	0/0 (0.00)	0/0 (0.00)	0/0 (0.00)
***P* value**	0.0583	>0.9999	0.0698	0.3153
**IGHD1**	1/3 (33.33)	0/4 (0.00)	1/3 (33.33)	1/5 (20.00)
**IGHD2**	2/4 (50.00)	0/4 (0.00)	1/4 (25.00)	1/5 (20.00)
**IGHD3**	4/13 (30.77)	3/16 (18.75)	0/15 (0.00)	1/18 (5.56)
**IGHD4**	1/4 (25.00)	1/4 (25.00)	1/4 (25.00)	0/5 (0.00)
**IGHD5**	0/2 (0.00)	0/3 (0.00)	0/2 (0.00)	0/3 (0.00)
**IGHD6**	1/3 (33.33)	1/5 (20.00)	1/4 (25.00)	1/6 (16.67)
**IGHD7**	0/0 (0.00)	0/0 (0.00)	0/0 (0.00)	0/0 (0.00)
**N/A**	0/2 (0.00)	1/4 (25.00)	0/3 (0.00)	0/4 (0.00)
***P* value**	0.9609	>0.9999	0.1133	0.5694
**IGHJ1**	0/1 (0.00)	1/1 (100.00)	0/1 (0.00)	1/1 (100.00)
**IGHJ2**	0/2 (0.00)	1/2 (50.00)	0/2 (0.00)	0/2 (0.00)
**IGHJ3**	1/3 (33.33)	1/3 (33.33)	1/3 (33.33)	0/4 (0.00)
**IGHJ4**	6/15 (40.00)	1/20 (5.00)	2/18 (11.11)	1/22 (4.55)
**IGHJ5**	2/4 (50.00)	0/6 (0.00)	1/4 (25.00)	0/7 (0.00)
**IGHJ6**	0/6 (0.00)	2/6 (33.33)	0/5 (0.00)	2/8 (25.00)
**N/A**	0/1 (0.00)	0/2 (0.00)	0/2 (0.00)	0/2 (0.00)
***P* value**	0.3566	0.0231*	0.5551	0.0737

The data was analyzed using the Fisher’s exact test with α<0.05 (two-sided). The asterisk (*) indicates that there is statistical significance (P value <0.05). P value < 0.05 indicates significant inter-group difference.

The intergroup differences among specific gene mutations and IGHV, IGHD, and IGHJ gene usage were analyzed using Fisher’s exact test with a<0.05 (two-sided). The asterisk (*) indicates that there is statistical significance (P value <0.05). P value < 0.05 indicates significant inter-group difference.Bold values indicate statistical significances (P value < 0.05).N/A, Not applicable.

## Discussion

During the process of B-cell maturation, the immunoglobulin genes in a B-cell undergo V(D)J recombination to produce a unique BCR for interaction with its specific antigen. The high proportion of stereotyped BCR major subsets (4, 5), the significantly biased BCR gene usage compared with the normal repertoire (26), and the remarkable effect of BTKs (27, 28) in CLL patients offer support for the theory that BCR signaling is involved in the tumorigenesis and development of CLL.

Several studies have stated explicitly that the subsets classified by IGH genes used in BCRs did differ in clinical courses and molecular features (3, 5, 29). These inspiring results have led some investigators to propose that these BCR components may help stratify CLL patients by risk level or identify groups with distinct clinical courses. The associations, mutual exclusivity, or differences among groups were analyzed through the collected information about cytogenetic and molecular aberrations and clinical profiles. The veracity of these statistics was validated by the strong correlation between cytogenetic aberrations and mutational status of the clonotypic IGHV gene (del11q and unmutated IGHV, del17p and unmutated IGHV, del13q and mutated IGHV, and complex karyotype and unmutated IGHV). Similarly, unfavorable gene mutations were significantly associated with unfavorable molecular prognostic factors (*NOTCH1* mutation and del17p, *TP53* mutation and del17p, *NOTCH1*and unmutated IGHV region). Surprisingly, the most frequent *IGLL5* mutation was significantly associated with a mutated clonotypic IGHV gene and seemed to be independent of known driving aberrations in CLL. *IGLL5* encodes an immunoglobulin lambda-like polypeptide. The second and third exons in *IGLL5* make up the immunoglobulin lambda joining 1 and the immunoglobulin lambda constant 1 gene segment (IGLJ1-C1). The sequencing analysis of IGH gene rearrangements used in our study did not cover immunoglobulin light chain genes. *IGLL5* was assumed to act as a possible independent driver mutation in lower-risk CLL (21). Several studies have supported the adverse effect of mutated *IGLL5*, such as the pathogenesis of MM (24) and Burkitt lymphoma (30), IGH translocations in CLL (25), and association with R/R DLBCL (23). However, some studies have also demonstrated that the overexpression of *IGLL5* affects immunological parameters, generating a more immune-suppressed tumor microenvironment (31). Thus, the conclusion on the *IGLL5* mutation is still ambiguous.

The associations between IGH genes used in BCRs and other factors in CLL were calculated. IGHJ3 genes were found to significantly correlate with mutated IGHV regions, while IGHJ6 genes were significantly associated with unmutated IGHV regions. The IGHJ6 gene was also reported to preferentially pair with unmutated IGHV and correlated with a shorter time to first treatment (TTT) in a previous study of CLL together with IGHD3-3 (32). Additionally, the IGHJ3 gene tended to co-occur with trisomy 12, while the IGHJ6 gene co-occurred with del17p more frequently. Based on these facts, we propose that the usage of IGHJ3 genes may help further support the prediction of a better prognosis, while IGHJ6 possibly tends to occur more frequently in cases with a worse prognosis. Patients expressing genes in the IGHD3 subgroup tended to carry more del13q aberrations than those expressing other IGHD genes, also indicating the likelihood of a favorable outcome. However, the use of IGHJ5, which was mutually exclusive with trisomy 12 and *NOTCH1* mutation, exerted a controversial effect on prognosis. The influence of HCDR3 length was also analyzed. The unmutated IGHV region was significantly associated with a longer HCDR3 region. IGHD2, IGHD3, and IGHD6 constituted longer HCDR3 regions than IGHD1 and IGHD5. IGHJ2 and IGHJ6 constituted significantly longer HCDR3 regions than IGHJ1, IGHJ3, IGHJ4, and IGHJ5, which again provides evidence for the potential positive effect of IGHJ3 and negative effect of IGHJ6. There is still a lack of studies that have directly focused on IGHJ genes. The usage of IGHJ genes is commonly discussed, accompanied by IGHV genes in stereotyped cases, such as IGHV1-69 in combination with IGHJ6 (32) or IGHJ3 (33). However, in our study, the partner IGHV genes of the IGHJ6, IGHJ3 or IGHJ5 gene varied in IGHV family clans, IGHV subgroups and genes. More experimental evidence is still needed to interpret our results on IGHJ genes since there are only 6 IGHJ genes compared to 45 IGHV genes. Whether the IGHJ gene, as a part of the HCDR3 region, also contributes to the risk stratification and prediction of prognosis in CLL is worthy of further discussion. Large-scale CLL studies containing information on IGH sequencing, other prognostic factors and follow-up survival time data will help clarify the exact correlation and corresponding mechanism.

There are still some limitations in our study. The first is the relatively small sample size due to the exclusion of patients who had incomplete or inconclusive IGH sequencing results. The cohort of patients was clinically heterogeneous at diagnosis (Binet A or Binet B-C), which could have influenced some of the results. Although significant associations or differences were found in the analysis of IGH genes and other prognostic factors in our study, more detailed analysis and validation may be accomplished in a study with a larger scale. Second, the statistical results in this study suggest that the IGHJ3 and IGHJ6 genes could have prognostic value; however, only a few studies have discussed the IGHJ genes themselves since these genes are not simply independent variables but are often associated with certain IGHV genes and their corresponding mutational status. One possible theory is that recombination signal sequences (RSSs) may impact the usage frequency of IGHJ by changing the lengths of the IGHJ6 spacer, the nonamer and heptamer of IGHJ3, and the nonamer of IGHJ5 (34). In contrast to IGHD gene usage, for which the utilization frequencies can be increased by deletion polymorphisms in the D locus, IGHJ gene usage was unaffected by polymorphisms of IGHD (35). Despite the inconclusive diagnostic value of IGHJ genes, they could be indirect indicators for prognosis. The low frequency of stereotyped BCR major subsets in our cohort is also intriguing. Studies on the IG repertoire of CLL demonstrated the existence of stereotyped BCR subsets with a frequency from 13.5% to 40% in patients (26); however, only two patients (2.82%) in our study were confirmed to carry a stereotyped BCR from subset #8 and subset #77. Analysis using the Arrest/assign Tool here only involved 19 major subsets that account for approximately 13.5% of stereotyped BCRs. We speculated that the frequency of stereotyped BCR subsets in total CLL cases differed among ethnic groups and regions (36–38) or that there existed more nonmajor subsets that were not identified by the Arrest/assign Tool. The last limitation is the missing data on clinical outcomes and follow-up visits due to loss to follow-up caused by the long time-scale and relatively indolent progression of most CLL cases. Possible longitudinal studies may help further delineate our conclusions.

In conclusion, by summarizing a series of information on cytogenetic and genetic aberrations and analyzing the frequency and distribution of IGH genes, we displayed an integrative and descriptive landscape of clinical and molecular profiles. Furthermore, the evaluation of the predictive value of IGH V(D)J recombination and the gene usage frequency was completed by analyzing the associations or mutual exclusivity among IGH genes and other clinical or molecular prognostic factors. We validated the favorable or unfavorable effects of the already known factors and further demonstrated the possible interactions of IGHV, IGHD, and IGHJ genes with these indicators and the involvement of IGH gene rearrangements in the initiation or development of CLL.

## Data availability statement

The data presented in the study are deposited in the SRA repository, accession number "PRJNA930566”. This project we created will be released in September 2023.

## Ethics statement

The studies involving human participants were reviewed and approved by Medical Ethics Committee of Tongji Hospital, Tongji Medical College of HUST (Approval number : TJ-IRB20200707). The ethics committee waived the requirement of written informed consent for participation.

## Author contributions

XD conceived, drafted the manuscript and drew the figures. MZ collected the statistics and discussed the manuscript. JW revised the manuscript. MX and XZ provided guidance and approved the version to be submitted. All authors contributed to the article and approved the submitted version.
