# Supplementary material for: Characterization of clonal immunoglobulin heavy V-D-J gene rearrangements in Chinese patients with chronic lymphocytic leukemia: Clinical features and molecular profiles
Source: Front Oncol. 2023 Feb 16;13:1120867. doi: 10.3389/fonc.2023.1120867 (PMC9978106; doi:10.3389/fonc.2023.1120867)
Supplement: Supplementary file 2 [file Table_2.docx]

**Supplementary Table 2. Sequencing panels used in the analysis of gene mutation**

| **Panel** | **Target** | **Type of sample** | **DNA input per sample** | **Sequencing library kit** | **Expected depth** | **Number of cases** |
| --- | --- | --- | --- | --- | --- | --- |
| **1**  **(CE, number of genes: 3)** | TP53, NOTCH1, SF3B1 | Fresh/Frozen BM | >100ng | / | / | 11 |
| **2**  **(number of genes: 40)** | ID3, NOTCH2, NRAS, PTEN, ATM, CCND1, KMT2D, KRAS, B2M, IDH2, CREBBP, CD79B, GNA13, STAT3, STAT5B, TP53, BCL2, KLF2, RPS15, TCF3, CD28, CXCR4, DNMT3A, SF3B1, PLCGI, MYD88, RHOA, FBXW7, TET2, PIMI, PRDMI, BRAF, CARD11, EZH2, MYC, CDKN2A, NOTCH1, BTK, DDX3X, PHF6 | Fresh/Frozen BM or FFPE | 20ng | Ion AmpliSeq™  Library Kit 2.0 | ＞500× | 3 |
| **3**  **(number of genes: 108)** | ALK, ASXLI, ATM, ATPбVIB2, B2M, BCL11B, BCL2, BIRC3, BRAF, BTK, CALR, CARD11, CBL, CCNDI, CCND2, CCND3, CCR4, CCR7, CD28, CD58, CD79B, CDKN2A, CEBPA, CNOT3, CREBBP, CRLF2, CSF1R, CSF3R, CXCR4, DNMT3A, EGFR, EP300, ETNK1, EZH2, FBXW7, FGFR3, FLT3, FOXOI, FYN, GATAI, GATA2, GATA3, GNAS, GNBI, ID3, IDH1, IDH2, ІКВKВ, IKZF3, IL7R, JAK1, JAK2, JAK3, KDMбA, KIT, KLF2, KMT2C, KRAS, MAP2KI, MAPKI, MED12, MEF2B, MPL, MYD88, NF1, NFKBIE, NOTCHI, NOTCH2, NPM1, NRAS, NT5C2, PAX5, PDGFRA, PHFó, PIMI, PKD1L2, PLCGI, PLCG2, POTI, POU2F2, PRKCB, PTEN, PTPNII, RHOA, RPL10, RPS15, RRAGC, RUNXI, SETBPI, SF3B1, SH2B3, SMARCA4, SMClA, SMC3, SOCSI, SRSF2, STAT3, STAT5B, STATб, TCF3, TNFRSF14, TP53, U2AF1, U2AF2, VMA21, WHSC1, WT1, XPO1 | Fresh BM | 15 ng for gDNA;  20ng for FFPE; | Ion AmpliSeq™  Library Kit 2.0 | ＞500× | 1 |
| **4**  **(number of genes: 129)** | ABL1, CTB, ARID1A, ATM, ATP6AP1, ATP6V1B2, B2M, BCL10, BCL2, BCL6, BCL7A, BIRC3, BMP7, BRAF, BTG1, BTG2, BTK, CARD11, CCND1, CCND3, CD19, CD22, CD58, CD70, CD79A, CD79B, CD83, CDKN2A, CIITA, CNOT3, CREBBP, CRLF2, CXCR4, DAZAP1, DDX3X, DTX1, DUSP2, EGFR, EGR1, EGR2, EIF2A, EP300, ETS1, ETV6, EZH2, FAS, FBXW7, FOXO1, GNA13, HIST1H1B, HIST1H1C, HIST1H1D, HIST1H1E, HNRNPH1, HVCN1, ID3, IDH1, IDH2, IGLL5, IKBKB, IKZF3, IL4R, IRF2BP2, IRF4, IRF8, ITPKB, KIT, KLF2, KLHL6, KMT2D(MLL2), KRAS, LTB, MAP2K1, MAP3K14, MED12, MEF2B, MS4A1(CD20), MYC, MYD88, NFKBIA, NFKBIE, NOTCH1, NOTCH2, NRAS, OSBPL10, P2RY8, PAX5, PCBP1, PIK3CA, PIK3CD, PIK3R1, PIM1, PLCG2, POT1, POU2AF1, POU2F2, PRDM1, PRKCB, PTEN, PTPN1, PTPRD, RHOA, RPS15, RRAGC, SF3B1, SGK1, SMARCA4, SOCS1, STAT3, STAT5B, STAT6, TBL1XR1, TCF3, TMEM30A, TMSB4X, TNFAIP3, TNFRSF14, TNFRSF17(BCMA), TP53, TRRAP, UBR5, VMA21, WHSC1, XPO1, ZFP36L1 | Fresh/Frozen BM or FFPE | >500ng | xGen® hybridization capture of DNA libraries | ＞500× | 6 |
| **5**  **(number of genes: 157)** | ABL1, ACTG1, AKT1, ARID1A, ATM, ATP6AP1, ATP6V1B2, B2M, BCL10, BCL11B, BCL2, BCL6, BCOR, BIRC3, BRAF, BTG1, BTG2, BTK, CARD11, CASP10, CCND1, CCND3, CCR4, CCR7, CD28, CD58, CD70, CD79A, CD79B, CD83, CDKN1B, CDKN2A, CHD2, CNOT3, CREBBP, CRLF2, CTNNB1, CXCR4, DDX3X, DIS3, DNM2, DNMT3A, DTX1, DUSP2, EBF1, EED, EGR1, EGR2, EIF2A, EP300, ETV6, EZH2, FAM46C, FAS, FAT1, FBXW7, FGFR3, FLT3, FOXO1, FYN, GATA3, GNA13, GNAQ, GPR183, HIF1A, HIST1H1B, HIST1H1C, HIST1H1D, HIST1H1E, HNRNPA2B1, HRAS, HVCN1, ID3, IDH1, IDH2, IGLL5, IKBKB, IKZF1, IKZF3, IL7R IRF4, ITPKB, JAK1, JAK2, JAK3, KDM6A, KIT, KLF2, KLHL6, KMT2C, KMT2D, KRAS, LTB, MAP2K1, MAP3K14, MAPK1, MAX, MED12, MEF2B, MYC, MYD88, NF1, NFE2, NFKBIE, NOTCH1, NOTCH2, NRAS, NT5C2, PAX5, PHF6, PIK3CA, PIK3R1, PIM1, PLCG1, PLCG2, POT1, POU2AF1, POU2F2, PRDM1, PRKCB, PTEN, PTPN1, PTPN11, RB1, RHOA, RPL10, RPS15, RRAGC, SAMHD1, SETD2, SF3B1, SGK1, SH2B3, SMARCA4, SMARCB1, SOCS1, STAT3, STAT5B, STAT6, TBL1XR1, TCF3, TET1, TET2, TMSB4X, TNFAIP3, TNFRSF14, TNFRSF1B, , TRAF3, TRRAP, U2AF1, USP7, VAV1, VMA21, WHSC1, WT1, XPO1 | Fresh/Frozen BM or FFPE | ≥ 50 ng for gDNA;  ≥ 100 ng for FFPE; | NanoPrep™ DNA Library Prep Kit for Illumina® | ＞500× | 25 |
