# Supplementary material for: Characterization of clonal immunoglobulin heavy V-D-J gene rearrangements in Chinese patients with chronic lymphocytic leukemia: Clinical features and molecular profiles
Source: Front Oncol. 2023 Feb 16;13:1120867. doi: 10.3389/fonc.2023.1120867 (PMC9978106; doi:10.3389/fonc.2023.1120867)
Supplement: Supplementary file 3 [file Table_3.docx]

**Supplementary Table 3. Correlation of IGH gene families and baseline clinical characteristics**

| **IGH**  **gene family** | **Rai Stage** (n=40),  n (I-IV, %) | **Binet Stage** (n=40),  n (B-C, %) | **Age** (n=78),  n (>65, %) | **Gender** (n=78),  n (male, %) |
| --- | --- | --- | --- | --- |
| **IGHV1** | 8/8 (100.00) | 5/7 (71.43) | 3/11 (27.27) | 11/11 (100%) |
| **IGHV2** | 3/3 (100.00) | 2/3 (66.67) | 2/3 (66.67) | 1/3 (33.33) |
| **IGHV3** | 16/17 (94.12) | 13/17 (76.47) | 22/39 (56.41) | 30/39 (76.92) |
| **IGHV4** | 9/10 (90.00) | 8/10 (80.00) | 8/22 (36.36) | 13/22 (59.09) |
| **IGHV5** | 0/0 (0.00) | 0/0 (0.00) | 1/1 (100.00) | 0/1 (0.00) |
| **IGHV6** | 1/1 (100.00) | 0/0 (0.00) | 0/2 (0.00) | 2/2 (100.00) |
| **Total** | 38 | 28 | 36 | 57 |
| **P value** | >0.9999 | >0.9999 | 0.1663 | **0.0158*** |
| **IGHD1** | 5/5 (100.00) | 5/5 (100.00) | 1/7 (14.29) | 6/7 (85.71) |
| **IGHD2** | 7/7 (100.00) | 5/6 (83.33) | 7/16 (43.75) | 12/16 (75.00) |
| **IGHD3** | 10/12 (83.33) | 8/11 (72.73) | 14/24 (58.33) | 18/24 (75.00) |
| **IGHD4** | 4/4 (100.00) | 2/4 (50.00) | 3/7 (42.86) | 6/7 (85.71) |
| **IGHD5** | 3/3 (100.00) | 2/3 (66.67) | 3/7 (42.86) | 4/7 (57.14) |
| **IGHD6** | 4/4 (100.00) | 3/3 (100.00) | 6/10 (60.00) | 7/10 (70.00) |
| **IGHD7** | 1/1 (100.00) | 0/1 (0.00) | 1/1 (100.00) | 0/1 (0.00) |
| **N/A** | 4/4 (100.00) | 3/4 (75.00) | 1/6 (16.67) | 4/6 (66.67) |
| **Total** | 38 | 28 | 36 | 57 |
| **P value** | 0.8923 | 0.4241 | 0.3032 | 0.7290 |
| **IGHJ1** | 2/2 (100.00) | 2/2 (100.00) | 1/2 (50.00) | 2/2 (100.00) |
| **IGHJ2** | 0/1 (0.00) | 0/1 (0.00) | 3/4 (75.00) | 3/4 (75.00) |
| **IGHJ3** | 5/6 (83.33) | 4/5 (80.00) | 5/10 (50.00) | 8/10 (80.00) |
| **IGHJ4** | 16/16 (100.00) | 12/14 (85.71) | 13/31 (41.94) | 22/31 (70.97) |
| **IGHJ5** | 8/8 (100.00) | 5/8 (62.50) | 6/13 (46.15) | 10/13 (76.93) |
| **IGHJ6** | 5/5 (100.00) | 4/5 (80.00) | 7/15 (7/15) | 10/15 (66.67) |
| **N/A** | 2/2 (100.00) | 1/2 (50.00) | 1/3 (33.33) | 2/3 (66.67) |
| **Total** | 38 | 28 | 36 | 57 |
| **P value** | **0.0269*** | 0.4426 | 0.9484 | 0.9887 |

The data was analyzed using Fisher’s exact test with α<0.05 (two-sided). *P* value < 0.05 indicates significant inter-group difference. The asterisk (*) indicates that there is statistical significance (*P* value ＜0.05).
