# Supplementary material for: Characterization of clonal immunoglobulin heavy V-D-J gene rearrangements in Chinese patients with chronic lymphocytic leukemia: Clinical features and molecular profiles
Source: Front Oncol. 2023 Feb 16;13:1120867. doi: 10.3389/fonc.2023.1120867 (PMC9978106; doi:10.3389/fonc.2023.1120867)
Supplement: Supplementary file 4 [file Table_4.docx]

**Supplementary Table 4. Distribution of gene mutations in CLL**

| **Gene**  **mutation** | **Mutation type**  (Number of cases with specific mutation type / total mutated cases) | **Number of cases**,  N / cases examined (%) |
| --- | --- | --- |
| **IGLL5** | Missense (5/9), nonsense (1/9), mutation in initial codon and splicing mutation (1/9), non-frameshift substitution (1/9), UTR mutation (1/9) | 9/31 (29.03) |
| **NOTCH1** | Frameshift deletion (5/6), nonsense (1/6) | 6/40 (15.00) |
| **MYD88** | Missense (2/4), non-frameshift substitution (1/4), nonsense (1/4) | 4/36 (11.11) |
| **TP53** | Missense (4/4) | 4/46 (8.70) |
| **POT1** | Missense (2/3), splicing (1/3) | 3/31 (9.68) |
| **KMT2D** | Nonsense (2/3), frameshift insertion (1/3) | 3/31 (9.68) |
| **EGR2** | Missense (3/3) | 3/31 (9.68) |
| **KRAS** | Missense (3/3) | 3/35 (8.57) |
| **NFKBIE** | Nonsense (1/2), frameshift deletion (1/2) | 2/32 (6.25) |
| **SAMHD1** | Missense (2/2) | 2/25 (8.00) |
| **SF3B1** | Missense (2/2) | 2/40 (5.00) |

**Supplementary Table 5.** **Correlation of number of mutated genes and prognostic markers**

| **Prognostic markers** | **Gene mutation number**, cases with specific feature/cases in each group (%) | | | | **P value** |
| --- | --- | --- | --- | --- | --- |
|  | **0** | **1** | **2** | **≥3** |  |
| **FISH** (n=22) |  |  |  |  |  |
| Tri(12) | 0/0 (0.00) | 0/7 (0.00) | 3/8 (37.50) | 1/7 (14.29) | 0.2699 |
| Del(11q) | 0/0 (0.00) | 0/7 (0.00) | 1/8 (12.50) | 0/7 (0.00) | >0.9999 |
| Del(13q) | 0/0 (0.00) | 4/7 (57.14) | 1/8 (12.50) | 2/7 (28.57) | 0.1944 |
| Del(17p) | 0/0 (0.00) | 0/7 (0.00) | 1/8 (12.5) | 2/7 (28.57) | 0.4909 |
| **U-CLL** (n=31) | 0/1 (0.00) | 3/9 (33.33) | 5/10 (50.00) | 3/11 (27.27) | 0.7658 |
| **CK** (n=20) | 0/0 (0.00) | 1/6 (16.67) | 1/7 (14.29) | 1/7 (14.29) | >0.9999 |
| **Age>65** (n=31) | 1/1 (100.00) | 3/9 (33.33) | 2/10 (20.00) | 7/11 (63.64) | 0.1079 |
| **Gender** (male, n=31) | 1/1 (100.00) | 5/9 (55.56) | 7/10 (70.00) | 9/11 (81.82) | 0.6285 |
| **Rai stage** (I-IV, n=15) | 0/0 (0.00) | 3/3 (100.00) | 7/7 (100.00) | 5/5 (100.00) | >0.9999 |
| **Binet stage** (B-C, n=15) | 0/0 (0.00) | 2/3 (66.67) | 6/7 (85.71) | 5/5 (100.00) | >0.9999 |

The data was analyzed using the Fisher’s exact test with α<0.05 (two-sided). The asterisk (*) indicates that there is statistical significance (*P* value ＜0.05).

**Supplementary Table 6. Correlation of number of mutated genes and prognostic markers**

| **Prognostic markers** | **Gene mutation number**,  cases with specific feature / cases in each group (%) | | | **P value** |
| --- | --- | --- | --- | --- |
|  | **0** | **1** | **≥2** |  |
| **FISH** (n=22) |  |  |  |  |
| Tri(12) | 0/0 (0.00) | 0/7 (0.00) | 4/15 (26.67) | 0.1866 |
| Del(11q) | 0/0 (0.00) | 0/7 (0.00) | 1/15 (6.67) | 0.6818 |
| Del(13q) | 0/0 (0.00) | 4/7 (57.14) | 3/15 (20.00) | 0.1069 |
| Del(17p) | 0/0 (0.00) | 0/7 (0.00) | 3/15 (20.00) | 0.2955 |
| **U-CLL** (n=31) | 0/1 (0.00) | 3/9 (33.33) | 8/21 (38.10) | >0.9999 |
| **CK** (n=20) | 0/0 (0.00) | 1/6 (16.67) | 2/14 (14.29) | 0.6807 |
| **Age>65** (n=31) | 1/1 (100.00) | 3/9 (33.33) | 9/21 (42.86) | 0.4282 |
| **Gender** (male, n=31) | 1/1 (100.00) | 5/9 (55.56) | 16/21 (76.19) | 0.5663 |
| **Rai stage** (I-IV, n=15) | 0/0 (0.00) | 3/3 (100.00) | 12/12 (100.00) | >0.9999 |
| **Binet stage** (B-C, n=15) | 0/0 (0.00) | 2/3 (66.67) | 11/12 (91.67) | 0.3714 |

The data was analyzed using the Fisher’s exact test with α<0.05 (two-sided). The asterisk (*) indicates that there is statistical significance (*P* value ＜0.05).

**Supplementary Table 7. Correlation of number of mutated genes and IGH gene families**

| **IGH**  **Gene families** | **Gene mutation number**,  Cases with specific feature / cases in each group (%) | | | |
| --- | --- | --- | --- | --- |
|  | 0 | 1 | 2 | ≥3 |
| **IGHV1** | 0/5 (0.00) | 1/5 (20.00) | 2/5 (40.00) | 2/5 (40.00) |
| **IGHV2** | 0/0 (0.00) | 0/0 (0.00) | 0/0 (0.00) | 0/0 (0.00) |
| **IGHV3** | 1/18 (5.56) | 7/18 (38.89) | 6/18 (33.33) | 4/18 (22.22) |
| **IGHV4** | 0/7 (0.00) | 1/7 (14.29) | 2/7 (28.57) | 4/7 (57.14) |
| **IGHV5** | 0/1 (0.00) | 0/1 (0.00) | 0/1 (0.00) | 1/1 (100.00) |
| **IGHV6** | 0/0 (0.00) | 0/0 (0.00) | 0/0 (0.00) | 0/0 (0.00) |
| **Total, n** | 1 | 9 | 10 | 11 |
| **P value** | >0.9999 | 0.6752 | >0.9999 | 0.1841 |
|  |  |  |  |  |
| **IGHD1** | 0/3 (0.00) | 1/3 (33.33) | 1/3 (33.33) | 1/3 (33.33) |
| **IGHD2** | 0/4 (0.00) | 1/4 (25.00) | 1/4 (25.00) | 2/4 (50.00) |
| **IGHD3** | 1/13 (7.69) | 4/13 (30.77) | 3/13 (23.08) | 5/13 (38.46) |
| **IGHD4** | 0/4 (0.00) | 1/4 (25.00) | 1/4 (25.00) | 2/4 (50.00) |
| **IGHD5** | 0/2 (0.00) | 1/2 (50.00) | 1/2 (50.00) | 0/2 (0.00) |
| **IGHD6** | 0/3 (0.00) | 1/3 (33.33) | 2/3 (66.66) | 0/3 (0.00) |
| **IGHD7** | 0/0 (0.00) | 0/0 (0.00) | 0/0 (0.00) | 0/0 (0.00) |
| **N/A** | 0/2 (0.00) | 0/2 (0.00) | 1/2 (50.00) | 1/2 (50.00) |
| **Total, n** | 1 | 9 | 10 | 11 |
| **P value** | >0.9999 | >0.9999 | 0.7543 | 0.7510 |
|  |  |  |  |  |
| **IGHJ1** | 0/1 (0.00) | 0/1 (0.00) | 0/1 (0.00) | 1/1 (100.00) |
| **IGHJ2** | 0/2 (0.00) | 1/2 (50.00) | 0/2 (0.00) | 1/2 (50.00) |
| **IGHJ3** | 0/3 (0.00) | 0/3 (0.00) | 1/3 (33.33) | 2/3 (66.67) |
| **IGHJ4** | 0/15 (0.00) | 5/15 (33.33) | 6/15 (40.00) | 4/15 (26.67) |
| **IGHJ5** | 0/4 (0.00) | 2/4 (50.00) | 0/4 (0.00) | 2/4 (50.00) |
| **IGHJ6** | 1/5 (20.00) | 1/5 (20.00) | 2/5 (40.00) | 1/5 (20.00) |
| **N/A** | 0/1 (0.00) | 0/1 (0.00) | 1/1 (100.00) | 0/1 (0.00) |
| **Total, n** | 1 | 9 | 10 | 11 |
| **P value** | 0.5000 | 0.8760 | 0.6646 | 0.4551 |

The data was analyzed using the Fisher’s exact test with α<0.05 (two-sided). The asterisk (*) indicates that there is statistical significance (*P* value ＜0.05).

**Supplementary Table 8. Correlation of specific gene mutation and clinical features**

| **Mutated**  **genes** | **Baseline Clinical features**, n, p | | | |
| --- | --- | --- | --- | --- |
|  | Age>65 | Gender, male | Rai stage, I-IV | Binet stage, B-C |
| **IGLL5** | 5, 0.4328 | 6, >0.9999 | 4, >0.9999 | 3, 0.4762 |
| **NOTCH1** | 2, 0.6726 | 5, >0.9999 | 4, >0.9999 | 4, >0.9999 |
| **MYD88** | 1, 0.6081 | 2, 0.5607 | 1, >0.9999 | 1, >0.9999 |
| **TP53** | 1, 0.6139 | 4, 0.5600 | 3, >0.9999 | 3, >0.9999 |

The data was analyzed using the Fisher’s exact test with α<0.05 (two-sided). The asterisk (*) indicates that there is statistical significance (*P* value ＜0.05).
